# Current State of Research Training in Pediatric Cardiology Fellowship Programs

**DOI:** 10.1007/s00246-025-03903-8

**Published:** 2025-06-25

**Authors:** Joseph R. Starnes, Rupali Gandhi, Laurie B. Armsby, Kerri A. Carter, Lowell H. Frank, Sinai C. Zyblewski

**Affiliations:** 1 Division of Pediatric Cardiology, Department of Pediatrics, Vanderbilt University Medical Center, 2200 Children’s Way, Nashville, TN 37233 USA; 2 Division of Pediatric Cardiology, Department of Pediatrics, Advocate Christ Medical Center, Oak Lawn, IL USA; 3 Division of Pediatric Cardiology, Department of Pediatrics, University of Chicago, Comer Children’s Hospital, Chicago, IL USA; 4 Division of Pediatric Cardiology, Department of Pediatrics, Oregon Health and Sciences University, Portland, OR USA; 5 Division of Pediatric Cardiology, Department of Pediatrics, Children’s Hospital of Richmond at VCU, Richmond, VA USA; 6 Division of Cardiology, Children’s National Hospital, Washington, DC USA; 7 Division of Pediatric Cardiology, Department of Pediatrics, Medical University of South Carolina, Charleston, SC USA

**Keywords:** Graduate medical education, Pediatrics, Cardiology, Research training

## Abstract

The Accreditation Council for Graduate Medical Education and the American Board of Pediatrics currently require a minimum of 12 months dedicated to research during pediatric cardiology fellowship training. Current data regarding the implementation of this requirement in pediatric cardiology fellowship programs are not available. We aimed to characterize current perceptions and practices of program directors regarding research supports, practices, and training in pediatric cardiology fellowship training programs. A web-based survey facilitated by the Society of Pediatric Cardiology Training Program Directors was sent to the 64 fellowship program directors in the USA. Data from responses were aggregated and reported. A total of 46 program directors responded to the survey for a response rate of 72%. Most programs (*n* = 39, 84.8%) included 12 months of research in their current curricula. Most programs included overnight call (*n* = 39, 84.8%), mandatory academic requirements (*n* = 42, 91.2%), and additional mandatory clinical responsibilities (*n* = 37, 80.4%) during research time. Many fellows pursued additional non-mandatory and elective clinical time during their research months. Most program directors (*n* = 29, 63.0%) thought the requirement for 12 months of research during the 36-month fellowship training period should be shortened. Most pediatric cardiology program directors believe the current 12-month research requirement should be shortened, with a goal of providing greater flexibility in training and a more individualized curriculum. Fellows wishing to perform additional research could utilize the additional elective rotations created by this change.

## Introduction

The Accreditation Council for Graduate Medical Education (ACGME) currently requires all pediatric subspecialty fellows to have a minimum of 12 months dedicated to research and scholarly activity during fellowship [1]. Fellows must complete a scholarly project under the supervision of a Scholarship Oversight Committee (SOC). Similarly, the American Board of Pediatrics (ABP) requires subspecialty fellows to have 12 months of dedicated research time, participate in a core research curriculum, engage in scholarly activity, and produce a work product prior to certification [2]. Examples of work products include peer-reviewed publications, manuscripts, theses, grants, and progress reports. These work products can be in basic, clinical, or translational research as well as health services, quality improvement, bioethics, education, or public policy. While the requirement for research activity comprises one-third of the fellowship training period, the vast majority of practicing pediatric cardiologists spend little of their professional time on research [3], with only about 3% spending more than half of their time conducting research [4].

In response to evolving workforce considerations and an increasing focus on mental health in pediatric primary care practices, the ACGME has recently released updated program requirements for pediatric residencies [5]. The modifications include a decrease in inpatient and critical care rotations in favor of more outpatient experiences and individualized curriculum time [5]. Similar changes have not been proposed for pediatric subspecialty training requirements. When surveyed, more than half of pediatric subspecialty fellows would have chosen to shorten their fellowship to two years by excluding the research component if the option were available [6].

Current data are not available regarding practices in research training in pediatric cardiology fellowship programs. We aimed to characterize current perceptions and practices of program directors regarding research supports, practices, and training in pediatric cardiology fellowship training programs. We hypothesized that program directors would be in favor of reduced research time requirements to allow for greater educational flexibility and individualized training. We further hypothesized that many fellows are currently using protected research time to pursue additional clinical experience.

## Methods

### Sampling and Survey

A secure web-based survey was facilitated by the Society of Pediatric Cardiology Training Program Directors (SPCTPD). Questions were drafted by SPCTPD leadership based on review of existing literature and were piloted internally prior to distribution. The survey was built using REDCap (Research Electronic Data Capture) tools [7, 8] and then distributed via email to all pediatric cardiology training program directors in the USA as of July 18, 2024. Periodic reminders were sent to program directors who had not yet responded. Questions were multiple-choice except for a free-text response allowing for additional comments.

### Statistical Analysis

Descriptive statistics were reported as counts and percentages for categorical variables and median with interquartile range (IQR) for continuous variables. Fisher’s exact and Wilcoxon rank-sum tests were used to compare values as appropriate. All analyses were performed using Stata version 14.2 (StataCorp LP, College Station, TX).

## Results

### Program Description

The survey was distributed to 64 program directors. A total of 46 program directors responded to the survey, representing a 72% response rate. Most programs (*n* = 28, 60.9%) had between four and nine categorical pediatric cardiology fellows (Table 1). A total of six (13.0%) programs had fewer than four fellows, and five programs (11.1%) had more than 15. Most programs had 12 research months built into their curricula. Most research months were scheduled later in fellowship with two (IQR 1,2) months in the first year and six (IQR 6,7) in the third year.

**Table 1:** Descriptive characteristics of programs

Total number of categorical fellows (*n* = 45)	
Less than 4	6 (13.3%)
4–6	15 (33.3%)
7–9	13 (28.9%)
10–12	4 (8.9%)
13–15	2 (4.4%)
16–18	2 (4.4%)
19 or more	3 (6.7%)
Total research months	
Less than 12 months	2 (4.4%)
12 months	39 (84.8%)
More than 12 months	5 (10.9%)
Research month distribution	
1st year	2 (1, 2)
2nd year	4 (3, 5)
3rd year	6 (6, 7)

Results presented as n (%) and median (IQR)

### Obligations During Research Time

Most programs (*n* = 39, 84.8%) scheduled fellows for overnight call during research time (Table 2). Similarly, most programs had additional academic requirements (*n* = 42, 91.3%), such as teaching and preparing surgical conferences, and other mandatory clinical responsibilities (*n* = 37, 80.4%) such as continuity clinic during research time. In aggregate, the additional academic and clinical responsibilities during research time accounted for less than 25% of fellows’ time for most programs (*n* = 29, 63.0%). Program directors reported that a substantial number of fellows chose to pursue additional elective clinical opportunities during their research time.

**Table 2:** Activities during research time

Clinical and academic responsibilities during research time	
Academic requirements^a^	42 (91.3%)
Night float	3 (6.5%)
Overnight call	39 (84.8%)
Other clinical responsibilities^b^	37 (80.4%)
Percent research time spent on mandatory clinical work (*n* = 37)	
Less than 25%	29 (63.0%)
25–50%	8 (17.4%)
Percent of time spent doing elective clinical work	
Less than 25%	29 (63.0%)
25–50%	11 (23.9%)
51–75%	5 (10.9%)
Greater than 75%	1 (2.2%)

^a^Presenting journal club, teaching responsibilities, and presenting/preparing surgical conference

^b^Continuity clinics, cross-coverage, and day shifts

### Research Resources and Output

**Fig. 1:**
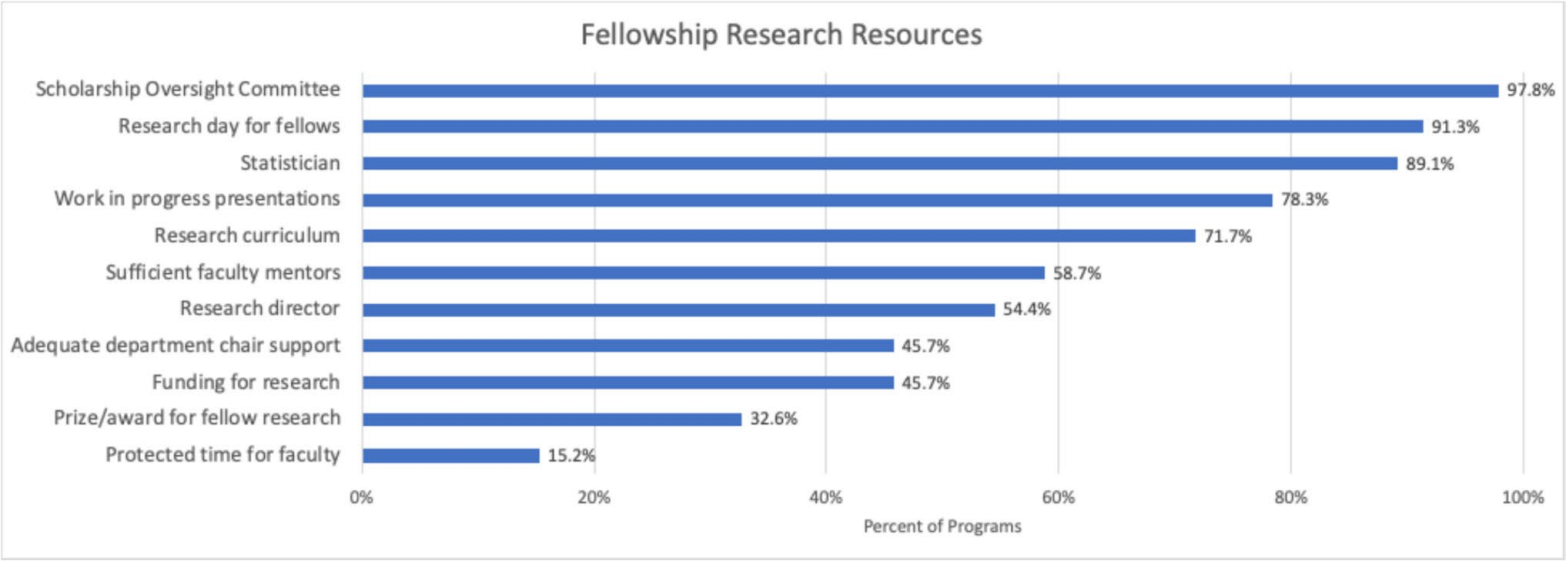
Fellowship research resources. Nearly, all programs offer Scholarship Oversight Committees, a research day for fellows, and statistical support. Very few programs have protected time for faculty to support fellow research or a research award for fellows. *n* = 46 for all resources

Nearly, all programs had SOCs, a research day for fellows, and a statistician to support fellow research (Fig. 1). More than 25% of programs did not have a formal research didactic curriculum, and nearly half of the programs reported insufficient availability of faculty mentors. Most programs did not have funding to support fellowship research and reported inadequate department chair support for fellow research. Only seven programs (15.2%) reported protected time for faculty to support fellow research.

Over half of program directors (*n* = 27, 58.7%) reported that more than 75% of graduating fellows completed a manuscript for submission to a peer-reviewed journal at or near the end of their categorical fellowship. Most program directors (*n* = 31, 67.4%) reported that the average number of submitted manuscripts was one per fellow. A smaller number (*n* = 9, 19.6%) reported 2–3 manuscripts per fellow. Nearly all program directors (*n* = 41, 89.1%) reported that fellows present abstracts at 1–3 national conferences during their categorical fellowship.

### Potential Requirement Changes

Most program directors supported a decrease in the current 12-month research requirement (*n* = 29, 63.0%), replacing it with an individualized curriculum tailored to the interests and needs of the trainee. None thought that the research requirements should be lengthened (Table 3). There was no difference in this perception between large programs (more than 15 fellows) and other programs (*p* = 0.441). Most program directors (*n* = 34, 73.9%) did not believe that shortening the research requirement would negatively impact the experience of scholarly activity during fellowship or the community of physician scientists in pediatric cardiology. Program directors suggested that additional research rotations could be included in the individualized curriculum for a fellow who desired or required additional time directed to scholarly activity.

**Table 3:** Perspectives on research requirement

Preference for 12-month requirement	
Shortened	29 (63.0%)
No change	17 (37.0%)
Lengthened	0 (0.0%)
Shortening will negatively impact physician scientists	
Yes	12 (26.1%)
No	34 (73.9%)
Requirement length preference	
No requirement	2 (4.4%)
3 months	3 (6.5%)
6 months	13 (28.3%)
9 months	12 (26.1%)
12 months	15 (32.6%)
Greater than 12 months	1 (2.2%)
Content of additional months (if selected < 12, n = 30)	
Required clinical months	7 (23.3%)
Elective clinical or research months	23 (76.7%)

### Comments

The survey included a free-text comments section, and 21 (45.7%) program directors supplied comments. Nearly all (17/21, 80.2%) commented on additional personalization of the fellowship curriculum. Many of these stated that fellows interested in research could pursue additional research time even if the requirement was shorter. At least nine of 21 (42.9%) mentioned shortening the research requirement, and none mentioned lengthening it.

## Discussion

We report current research training practices, supports, and productivity among nearly 75% of pediatric cardiology training programs in the USA. Our study identified a misalignment between ACGME requirements, research training resources and practices, and workforce trends. Essentially all programs have mandatory clinical and academic responsibilities during research time, and many fellows pursue additional elective clinical time during these weeks. Research resources are limited at many programs, including inadequate faculty mentorship and lack of funding support. Most program directors thought that the current 12-month research requirement should be shortened and replaced with an individual curriculum tailored to the needs of each trainee.

Our data provide the first description of the current national implementation of research requirements in pediatric cardiology fellowship programs. The vast majority of programs meet but do not exceed the requirement with exactly 12 months of research in their curriculum. Research months are more common in the second and third years of fellowship, consistent with a heavy clinical workload during the first year in most programs. The large portion of time allotted to research, including half of the third year, is in contrast to the 3% of practicing pediatric cardiologists who report spending more than half of their time on research [4]. The varied and increasing clinical settings in which fellows require training (cardiac critical care, echocardiography, interventional cardiology, electrophysiology, etc.) has increased pressure on the 24 months of clinical training during fellowship. Fellows seek out additional clinical experiences to further their mastery of clinical cardiology and to better prepare themselves for the workforce. This is consistent with the fact that most fellows now pursue additional and advanced subspecialty clinical training, mostly through fourth-year fellowships [3]. The increase in fourth-year training is driven in part by the increasing complexity within these clinical disciplines. Additionally, much of the research time currently incorporated into fellowship is in the second and third years of training. Significant research time late in the third year may lead to a lack of clinical exposure immediately prior to the transition to the attending role, leaving fellows underprepared for this transition if entering practice after three years.

There were significant gaps in resources available to support fellow research. Nearly 30% of programs reported not having a structured research curriculum despite this being an ABP requirement for board certification [2], as well as correlated with increased productivity without increasing the amount of research time [9, 10]. Additionally, there are notable deficits in faculty and financial support of fellow research. Although research mentorship has been found to be an important factor in research productivity both in pediatric residencies [11] and fellowships [12], more than 40% of programs report insufficient faculty mentorship, and only seven programs report protected time for faculty to provide mentorship. Finally, fewer than half of programs reported funding for fellow research. Accrediting bodies should consider formal criteria for the support for fellow research afforded by programs and institutions.

Our findings are consistent with a recent survey of pediatric cardiology fellows [13]. This study found that just 31% of fellows were satisfied with the research methodological training they have received in fellowship. Like our study, they identified lack of protected time for faculty to support fellow research, lack of formal research training, and lack of funding for fellow research as key barriers. Interestingly, they identified that 26% of fellows reported having nine months or less of protected research time. This contrasts with the 4% of program directors in our survey, although these numbers are not directly comparable as multiple fellows could have been from the same program. This survey of fellows did not directly address whether fellows would prefer shorter research requirements.

Most fellowship program directors thought the current 12-month research requirement should be shortened, and most did not think this would affect the overall productivity of scholarly activity or the community of physician scientists. This is aligned with the preference of most fellows who indicate they would shorten their fellowship by shortening the research requirements if the option were available [6]. Interestingly, there is no ACGME research time requirement for adult cardiology fellowships [14]. Although not formally analyzed in this study, the desire for shorter research requirements was echoed by written comments in the survey. It was generally thought that if the research requirement were shortened, those interested in research could utilize additional elective opportunities to expand their work, while those not interested in research could seek out other opportunities relevant to their clinical goals. Many were in favor of tracks within programs that would allow research- or clinically focused fellows to pursue different allocations of their training time. Such changes would be in line with the recent update to the ACGME pediatric residency requirements, which emphasize flexibility and individualization [5]. Additional rotations individualized to the trainee’s goals may allow some fellows to acquire advanced clinical skills and experience during their three-year categorical fellowship that would normally require a fourth-year fellowship. Further, the increasing clinical complexity in pediatric cardiology, together with the recent ACGME changes to pediatric residency, have substantially increased the challenge of providing adequate training within the clinical training portion of fellowship. With changes in residency training, incoming fellows will have less inpatient clinical experience and some of that training will now need to occur in fellowship. Importantly, physicians across specialties show limited ability to assess their competency [15]. While many fellows express a desire to shorten fellowship by decreasing research time [6], using this for additional elective clinical or research time based on interest may provide more robust training.

### Limitations

We are limited in part by the potential for response bias, including that those who are satisfied with current research requirements may be less likely to respond. The effect of this on the findings is likely minimal given the relatively high response rate. As in all survey research, there is also the potential for social desirability bias. It is possible that available resources are more limited than reported. Finally, we report the responses of program directors and not fellows themselves. It is possible that fellows may report different preferences regarding protected research time and the desired length of the research requirement. This is an interesting area for potential research. Further, program directors who are new to their role or have limited direct engagement with fellows may not fully understand the impact of research training on the fellowship experience.

## Conclusion

Nearly all pediatric cardiology fellowship programs schedule mandatory clinical and academic responsibilities during research time, and many fellows pursue additional elective clinical activities during this time. Research resources are limited at many programs, including inadequate faculty mentorship and lack of funding support. Most pediatric cardiology program directors believe the current 12-month research requirement should be shortened in order to allow for an individualized curriculum tailored to the needs of each trainee and to better prepare graduates for the work they will be doing in their career.

## Data Availability

The datasets used and analyzed during the current study are available from the corresponding author on reasonable request.
